# Systematic review and meta-analysis of postoperative pain and symptoms control following laser haemorrhoidoplasty versus Milligan-Morgan haemorrhoidectomy for symptomatic haemorrhoids: a new standard

**DOI:** 10.1007/s00384-022-04225-4

**Published:** 2022-07-29

**Authors:** Varen Zhi Zheng Tan, Ern-wei Peck, Sharmini S. Sivarajah, Winson J. Tan, Leonard M. L. Ho, Jia-Lin Ng, Cheryl Chong, Darius Aw, Franky Mainza, Fung-Joon Foo, Frederick H. Koh

**Affiliations:** 1 Yong Loo Lin School of Medicine, National University of Singapore, Singapore, Singapore; 2 Colorectal Service, Division of Surgery, Sengkang General Hospital, SingHealth Services, Singapore, Singapore; 3 Pondok Indah Hospitals Group, Jakarta, Indonesia

**Keywords:** Haemorrhoids, Laser, Technique, Surgery, Outcomes

## Abstract

**Purpose:**

Haemorrhoidal disease (HD) plagues one in every ten people, with a plethora of surgical treatment modalities, of which laser haemorrhoidoplasty (LHP) is a relatively novel option. This systematic review and meta-analysis objectively evaluated the efficacy, safety, and tolerability of LHP compared against conventional (Milligan-Morgan) open haemorrhoidectomy (CoH).

**Method:**

A comprehensive search of MEDLINE, EMBASE, CENTRAL, and Google Scholar was conducted. Randomised controlled trials (RCTs) and comparative cohort studies (CCSs) which compared LHP against CoH were included**,** with postoperative pain as the primary outcome. Secondary outcomes included intraoperative characteristics, short- and moderate-term outcome, and complications.

**Results:**

A total of 12 studies (6 RCTs and 6 CCSs), with a total of 1824 patients, were analysed. LHP resulted in reduced postoperative pain for the first day (mean difference of 2.07 visual analogue scale units), week, and month. The mean dosage and duration of postoperative analgesia use was similarly lower, with a mean difference of 4.88 mg (morphine) and 2.25 days, respectively. Crucially, recurrence was equivocal (HR: 0.72, CI: 0.21–2.40) at a mean follow-up duration of 8.58 ± 9.55 months. LHP resulted in lower blood loss and was 12.74 min shorter on average. LHP’s postoperative recovery time was 9.03 days less with equivalent or decreased risk of most short- and moderate-term complications except anal thrombosis.

**Conclusion:**

Our study suggests that LHP is more tolerable than CoH, providing patients with superior postoperative quality of life at equivalent moderate-term efficacy. These findings contribute to improved understanding of LHP and its potential at enhancing the quality of HD care.

**Supplementary Information:**

The online version contains supplementary material available at 10.1007/s00384-022-04225-4.

## Introduction

HD is widely prevalent, plaguing up to 11% of the population [1–3]. The disease is defined by the abnormal dilatation and distortion of vasculature with subsequent connective tissue destruction within the anal cushion [4]. The aetiopathogenetic theory concerning HD is multifactorial and complex; factors contributing to its pathogenesis are manifold. It is mostly thought to arise from vascular tone dysregulation and vascular hyperplasia, contributed by elevated intraabdominal pressure and increased inflow of the superior rectal artery, ultimately causing dilatation of the haemorrhoidal plexus. Whilst benign, it causes marked discomfort, anxiety, and distress [5, 6]. Management depends on patient factors and grading; [5, 7] surgery is usually indicated after failure of conservative measures or higher grades (III and IV), classified by grading scales such as the Banov, Goligher, or BPRST classification [8–10].

Conventional open haemorrhoidectomy (CoH), initially described by Milligan-Morgan, is still regarded by literature in the modern era as the current gold standard surgical treatment [11, 12]. It is adopted globally and provides low recurrence, where haemorrhoids are excised with a scalpel [13]. Unfortunately, it is associated with significant postoperative pain and risk of postoperative complications [14]. Alternative operations such as the Ferguson closed haemorrhoidectomy, rubber-band ligation, and stapled haemorrhoidopexy were subsequently developed in efforts to mitigate said complications associated with CoH [15–19] but they were found to be compromised by pelvic sepsis, postoperative bleeding, and higher recurrence [20–22].

Non-excisional laser haemorrhoidoplasty (LHP) is a relatively novel minimally invasive modality, comprising of laser probe introduced through a small incision at the ano-cutaneous junction and anodermis into the haemorrhoid [23]. Radial energy at a wavelength of 980 to 1470 nm is deployed from the laser diode into the haemorrhoid cushion. Thermal energy causes closure of the haemorrhoidal plexus by venous thrombosis and obliteration of downstream haemorrhoidal cushions, with adherence of the rectal mucosal and submucosal layers to the underlying muscular layer whilst avoiding injury to the inner lining of the anal canal. This initiates fibrosis and tissue remodelling, causing volume reduction and eventual obliteration of the haemorrhoidal tissue [24–27]. An anorectal mucopexy can also be performed in the same setting with absorbable sutures to hitch up any remaining prolapse after the laser coagulation.

LHP and CoH are conducted under general or spinal anaesthesia, typically in ambulatory surgical settings, with oral analgesia for postoperative pain management. Previous studies have demonstrated reduced postoperative pain and risk of bleeding post-LHP, [27–31] recommending it for grade II and III HD with satisfactory long-term outcomes compared to CoH [24, 32]. Consequently, this study was conducted to evaluate the efficacy, safety, and tolerability of LHP compared against the established standard surgical modality that is CoH. This systematic review identified all existing randomised controlled trials (RCTs) and prospective comparative cohort studies (CCSs) and conducted a pairwise meta-analysis primarily to compare postoperative pain, with secondary objectives, including efficacy, clinical outcomes, and complication rates.

## Materials and methods

### Search strategy

A systematic literature review was conducted for all RCTs and CCSs comparing LHP against CoH in accordance with the Preferred Reporting Items for Systematic Reviews and Meta-Analyses (PRISMA) guidelines [33]. Articles published in MEDLINE/Pubmed, Embase, and Cochrane Central Register of Controlled Trials (CENTRAL) in the Cochrane Library were identified using search terms ‘Haemorrhoid’ (MeSH term), ‘Laser’, ‘Open’, ‘Milligan-Morgan’, ‘Conventional’, ‘Excisional’, ‘Haemorrhoidectomy’ (MeSH term), and ‘Clinical Trial’ (MeSH term). The top 100 most relevant results from Google Scholar were screened for each search string, in concordance with recommendations as an adjunctive database [34–36]. The references of shortlisted articles were searched. The last search date was 27th June 2021. The study protocol was registered in PROSPERO, the international prospective register of systematic reviews, on 31st July 2021 (CRD42021271029), with no post-registration amendments.

### Inclusion and exclusion criteria

Original RCTs and CCSs comparing LHP against CoH were considered for this meta-analysis. Studies that compared LHP to other modalities were included if a comparison was made with CoH. No restrictions were made based on publication year or article language. All other study types were excluded.

### Outcomes of interest

Data from individual studies were tabulated, including study design, demographical, and clinical parameters of patients, HD grade according to the Banov grading scale, and procedural details. Table 1 summarises the characteristics of included studies. The primary and secondary outcomes of this study were defined based on the repeatedly reported disadvantages of postoperative pain and complications and the advantage of low disease recurrence associated with CoH, to objectively assess and compare LHP against it.

**Table 1:** Characteristic of included studies

S/N	Reference	Study period	Country of study	Number of centres	Study type	Duration of follow-up (months)	Laser wavelength (nm)	Laser device	Primary outcome	Secondary outcomes	Intervention		
											LHP	EH	Total
1	Nazari MS et al. [41]	2010–2011	Iran	-	RCT	6	980	-	Mean pain on postoperative day 1	• Intraoperative and postoperative bleeding • Urinary retention • Wound infection • Length of hospitalisation • Administered morphine dose	29	30	59
2	Naderan M et al. [59]	2011–2012	Iran	1	RCT	12	980	Biolitec AG CeramOptec	Mean pain on postoperative day 1	• Intraoperative and postoperative bleeding • Urinary retention • Wound infection • Administered morphine dose • Operating time • Recurrence	30	30	60
3	Alsisy AA et al. [58]	2016–2017	Egypt	1	RCT	3	980	A.R.C Laser Gmbh	Mean pain on postoperative day 1	• Intraoperative and postoperative bleeding • Urinary retention •Wound infection • Administered morphine dose • Operating time • Recurrence	30	30	60
4	Eskandaros MS et al. [52]	2017–2019	Egypt	1	RCT	12	1470	Cerlas Diode Laser (Biolitec)	Mean pain on postoperative day 1, weeks 1, 2, and 3, and months 1 and 2	• Operating time • Length of hospitalisation • Return to daily activities • Urinary retention • Postoperative bleeding • Anal stenosis • Recurrence	40	40	80
5	Shabahang H et al. [44]	2011–2013	Iran	2	RCT	6	1470	Cerlas Diode Laser (Biolitec)	Mean pain on postoperative day 1	• Postoperative bleeding • Urinary retention • Painful defecation • Wound infection • Anal stenosis, fistula, and thrombosis • Fecal incontinence • Length of hospitalisation • Quality of life of patients 6 months after operation based on SF-36 questionnaire	40	40	80
6	Poskus T et al. [43]	2016–2018	Lithuania	1	RCT	12	1470	Cerlas Diode Laser (Biolitec)	Recurrence of rectal bleeding and prolapse at 1 year after operation	• Time to return to work or normal activity • Mean pain on postoperative day 1 to 7 • Fecal incontinence • Quality of life of patients 6 months after operation based on SF-36 questionnaire • Recurrence	40	40	80
7	Plapler H et al. [60]	2009	Brazil	1	CCS	1	810	Synus Laser Inc	Mean pain on postoperative day 1, week 1, and month 1		15	10	25
8	Maloku H et al. [57]	2012–2014	Kosovo	1	CCS	2	980	Biolitec	Mean pain on postoperative day 1, weeks 1, 2, and 3, and months 1, 2, and 6	• Operating time	20	20	40
9	Maloku H et al. [53]	2014–2015	Montenegro	1	CCS	6	980	Biolitec	Mean pain on postoperative day 1, weeks 1, 2, and 3, and months 1 and 2	• Postoperative bleeding • Length of hospitalisation • Recovery time • Operating time	100	100	200
10	Mohammed AF et al. [54]	2014–2018	Iraq	1	CCS	36	980	-	Mean pain on postoperative day 1, weeks 1, 2, and 3, and months 1, 2, and 6	• Postoperative bleeding • Wound infection • Anal stenosis • Recurrence	500	500	1000
11	Hassan AM et al. [56]	2019–2020	Egypt	1	CCS	6	1470	Biolitec	Mean pain on postoperative day 1, weeks 1, 2, and 3, and months 1, 2, and 6	• Operating time • Length of hospitalisation • Postoperative bleeding, pain, abscess, oedema • Anal fistula and stricture • Faecal incontinence • Recurrence	20	20	40
12	Khan HM et al. [55]	2019–2020	India	1	CCS	1	1470	Lasotronix	Mean pain on postoperative day 1, weeks 1, 2, and 3, and month 1	• Intraoperative and postoperative bleeding • Operating time • Administered morphine dose • Length of hospitalisation • Recovery time • Urinary retention • Anal stenosis, thrombosis and discharge	50	50	100
											**914**	**910**	**1824**

The primary outcome assessed was postoperative pain, measured with the visual analogue scale (VAS) on days 1, 7, and 1 month after surgery, as all included studies reported according to this timeline to provide common timepoints of comparison. Postoperative pain was also measured indirectly through analgesia dose, duration of usage, and its post-discharge usage. Secondary outcomes included intraoperative characteristics, postoperative short- and moderate-term outcome, and complications. Postoperative short-term outcomes included were duration of hospitalisation and recovery time, defined as the time required to return to work or normal activity. Short-term complications included acute urinary retention (ARU), significant postoperative bleeding, reoperation, early recurrence, anal thrombosis, and acute anal discharge. Moderate-term outcomes included recurrence and assessment of postoperative quality of life (QoL). Recurrence was defined as recurrent internal or prolapsed haemorrhoids at the studies’ maximum follow-up period. Postoperative QoL was partly assessed by standardised questionnaires. Moderate-term complications included anal stenosis, bowel incontinence, and perianal skin tags.

### Data extraction and synthesis

Relevant studies were identified by two authors through title and abstract screening. Further selection was based on full text. Any discrepancy was resolved by the senior author after discussion. Aforementioned data parameters were collected independently through a predetermined standardised data extraction form. Risk of bias assessment was performed using the revised Cochrane risk-of-bias tool for randomised trials (RoB 2) for RCTs and the risk of bias in non-randomised studies of interventions (ROBINS-I) for CCSs independently [37, 38].

### Statistical analysis

Data were presented as mean with standard deviation, and frequencies as appropriate. Comparison between patients who underwent LHP (LHP group) and CoH (CoH group) were performed using chi-square test for categorical outcomes. A confidence level of *p* < 0.05 was considered statistically significant.

For each outcome, forest plots were rendered to include all applicable studies. Begg’s funnel plot was drawn for postoperative day 1 pain to assess publication bias [39] with Egger’s regression test. The presence of heterogeneity was assessed using the *I*^*2*^ index. The fixed-effects model was utilised for plots with *I*^*2*^ index less than 70% and the random-effects model for those more than 70% [40]. For binary data, a binomial model was used for the analysis, and the hazards ratio (HR) was calculated. The mean difference (MD), confidence interval (CI), and p-value were calculated for continuous outcomes [41].

All analyses were performed using the Review Manager (RevMan) computer program (version 5.4. Copenhagen, Denmark: The Nordic Cochrane Centre, The Cochrane Collaboration, 2020), GraphPad Prism v9 (GraphPad Software, La Jolla California, USA), Comprehensive Meta-Analysis Version 2 (Engelwood, NJ, Biostat; 2005), Risk-of-bias VISualization (robvis): An R package, and Shiny web app for visualising risk-of-bias assessments [42].

## Results

### Eligible studies

**Fig. 1:**
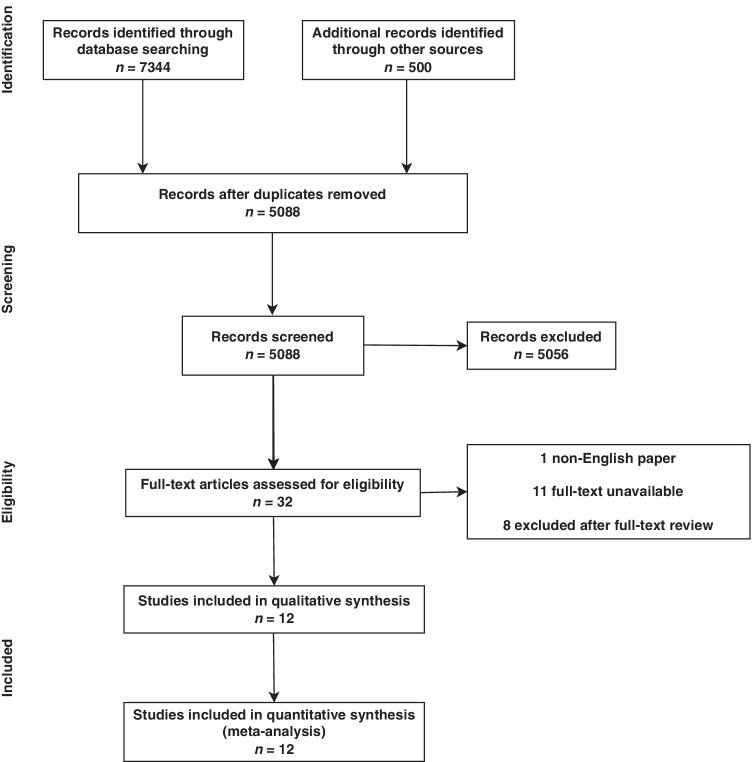
PRISMA flowchart

The initial search revealed a total of 7844 studies, of which 2756 were duplicates and 5088 were original studies. Non-relevant articles were excluded based on title and abstract screening, resulting in 32 studies. After examination by full text, 20 studies were excluded, leaving 12 studies (Fig. 1), of which 6 (50%) were RCTs with 419 (23%) total patients, and 6 (50%) studies were non-randomised, with 1405 (77%) total patients. The total cohort from all included studies was 1824 patients. The mean duration of follow-up was 8.58 ± 9.55 months. Sample sizes for individual studies ranged from 25 to 1000 patients (Table 1).

### Demographic characteristics

The mean patient age ranged from 34.7 to 47.0 years for the LHP group and 33.7 to 49.0 years for the CoH group. With the exclusion of data from Plapler et al. [43] as gender was not reported, a male preponderance of 65.3% (*n* = 1174/1799) was observed in the total cohort. LHP group had 65.0% males (*n* = 584/899) and CoH group had 66.1% males (*n* = 588/900), with no statistical difference between them (*p* = 0.87). With the exclusion of data from Nazari et al. as grade breakdown was not specified, [44] most patients had Banov grade II or III haemorrhoids (97.6%, *n* = 1722/1765), with none having grade I haemorrhoidal disease (Table 2). The most common indications for surgery were bleeding (76.1%, *n* = 350/460), pain (37.4%, *n* = 172/460), and itching (17.4%, *n* = 80/460). The CoH group had a higher proportion of patients presenting with itching (*p* = 0.02).

**Table 2:** Characteristics of study participants from included studies

Reference	Study type	Interventional arm	Number of participants, *n* (%)	Mean age, years ± SD	Sex, *n* (%)		Haemorrhoid grade, *n* (%)
					M	F	
Nazari MS et al. [41]	RCT	LHP	29 (49.15)	43.3 ± 13.8	19 (65.5)	10 (34.5)	All grade III or IV
		CoH	30 (50.85)	47.2 ± 14.0	12 (40.0)	18 (60.0)	All grade III or IV
Naderan M et al. [59]	RCT	LHP	30 (50.0)	43.7 ± 13.7	13 (43.3)	17 (56.7)	II: 13 (43.3) III: 17 (56.7)
		CoH	30 (50.0)	44.3 ± 11.3	11 (36.7)	19 (63.3)	II: 10 (33.3) III: 20 (66.7)
Alsisy AA et al. [58]	RCT	LHP	30 (50.0)	34.73 ± 10.17	18 (60.0)	12 (40)	II: 13 (43.3) III: 17 (56.7)
		CoH	30 (50.0)	33.67 ± 10.22	15 (50)	15 (50)	II: 17 (56.7) III: 13 (43.3)
Shabahang H et al. [44]	RCT	LHP	40 (50.0)	38.12 ± 8.29	18 (45.0)	22 (55.0)	II: 29 (72.5) III: 11 (27.5)
		CoH	40 (50.0)	38.72 ± 9.52	18 (45.0)	22 (55.0)	II: 25 (62.5) III: 15 (37.5)
Eskandaros MS et al. [52]	RCT	LHP	40 (50.0)	40.8 ± 8.8	27 (67.5)	13 (32.5)	III: 40 (100.0)
		CoH	40 (50.0)	41.0 ± 8.8	29 (72.5)	11 (27.5)	III: 40 (100.0)
Poskus T et al. [43]	RCT	LHP	40 (50.0)	47.0 ± 13.0	27 (67.5)	13 (32.5)	II: 10 (25.0) III: 30 (75.0)
		CoH	40 (50.0)	45.0 ± 12.0	19 (47.5)	21 (52.5)	II: 7 (17.5) III: 33 (82.5)
Plapler H et al. [60]	CCS	LHP	15 (60.0)	n.r	n.r	n.r	All grade II or III
		CoH	10 (40.0)	n.r	n.r	n.r	All grade II or III
Maloku H et al. [57]	CCS	LHP	20 (50.0)	47.0 ± 12.6	11 (55.0)	9 (45.0)	III: 20 (100.0)
		CoH	20 (50.0)	49.0 ± 12.3	12 (60.0)	8 (40.0)	IV: 20 (100.0)
Maloku H et al. [53]	CCS	LHP	100 (50.0)	47.0 ± 12.6	57 (57.0)	43 (43.0)	III: 100 (100.0)
		CoH	100 (50.0)	49.0 ± 12.3	64 (64.0)	36 (36.0)	III: 100 (100.0)
Mohammed AF et al. [54]	CCS	LHP	500 (50.0)	n.r	350 (70.0)	150 (30.0)	All grade II or III
		CoH	500 (50.0)	n.r	368 (73.6)	132 (26.4)	All grade II or III
Hassan AM et al. [56]	CCS	LHP	20 (50.0)	47.0 ± 12.6	12 (60.0)	8 (40.0)	III: 20 (100.0)
		CoH	20 (50.0)	49.0 ± 12.3	11 (55.0)	9 (45.0)	III: 20 (100.0)
Khan HM et al. [55]	CCS	LHP	50 (50.0)	42.7 ± 10.1	32 (64.0)	18 (36.0)	III: 27 (54.0) IV: 23 (46.0)
		CoH	50 (50.0)	41.6 ± 10.3	29 (58.0)	21 (42.0)	III: 30 (60.0) IV: 20 (40.0)

### Methodological quality of included studies

The RoB 2 and ROBINS-I revealed all six RCTs of ‘low risk’ and all six CCSs to be of ‘moderate risk,’ respectively. Egger’s test confirmed no significant publication bias: *t* = 1.69 (95% CI =  − 31.5 to 7.9; *p* = 0.17).

### Postoperative pain and analgesia

**Fig. 2:**
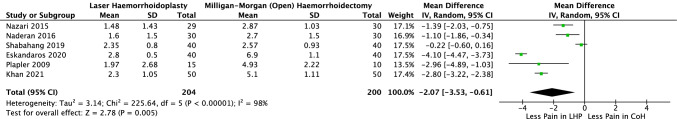
Forest plot of mean pain (postoperative day 1)

**Fig. 3:**
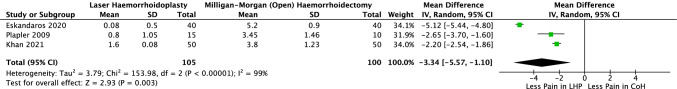
Forest plot of mean pain (postoperative week 1)

**Fig. 4:**
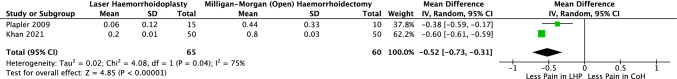
Forest plot of mean pain (postoperative month 1)

A total of 6 studies reported pain through the VAS on postoperative day 1 with 404 patients. The LHP group had significantly reduced pain compared to the CoH group with a mean difference of 2.07 (CI: 0.61–3.53, *p* = 0.005) units (Fig. 2). Pain at postoperative week 1 (Fig. 3) and month 1 (Fig. 4) demonstrated congruent findings, with a mean difference of 3.34 (CI: 1.10–5.57, *p* = 0.003) units and 0.52 (CI: 0.31–0.73, *p* < 0.0001) units of 3 and 2 studies (*n* = 205 and *n* = 125), respectively.

**Fig. 5:**
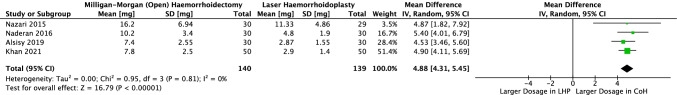
Forest plot of mean dosage of analgesia

There was a reduction in the mean dosage of analgesia used postoperatively in the LHP group compared to the CoH group, with a mean difference of 4.88 mg (CI: 4.31–5.45, *p* < 0.0001) of morphine, reported by 4 studies (*n* = 279) (Fig. 5). Similarly, 2 studies (*n* = 140) observed a shorter duration of post-discharge use of oral analgesia with a mean difference of 2.25 days (CI: 0.83–3.67, *p* = 0.002) favouring the LHP group. A total of 2 studies (*n* = 120) demonstrated no significant difference in the number of patients who used post-discharge oral analgesia.

### Intraoperative characteristics

A total of 9 studies (*n* = 719) reported the mean duration of surgery, where LHP was shorter than CoH, with a mean difference of 12.74 min (CI: 10.05–15.43, *p* < 0.0001). Additionally, the pooled estimate from 4 studies (*n* = 279) demonstrated that LHP resulted in less intraoperative blood loss, a mean difference of 16.43 ml (CI: 9.05–23.82, *p* < 0.0001).

### Short-term outcome and complications

The short-term outcome was overall better after LHP. 4 studies (*n* = 320) described the mean duration of hospitalisation, which demonstrated no significant difference but tended to favour LHP with a mean difference of 0.45 days (CI: − 0.14–1.03, *p* = 0.13). A total of 4 studies (*n* = 440) revealed a shorter mean recovery time after discharge in the LHP group, with a mean difference of 9.03 days (CI: 1.87–16.18, *p* = 0.01).

From the analysis of 7 studies (*n* = 1540), LHP demonstrated a significantly lower risk ratio (RR) of significant postoperative bleeding at 0.22 (CI: 0.14–0.36, *p* < 0.0001). Similarly, though not significant, 2 studies (*n* = 160) demonstrated that LHP patients were less likely to have anal discharge with a HR of 0.13 (CI: 0.02–0.98, *p* = 0.05). The pooled estimate from 6 studies (*n* = 1359) demonstrated no significant difference in the RR of developing ARU; however, it tended to favour LHP at 0.23 (CI: 0.05–1.15, *p* = 0.07). On the other hand, there was a significantly elevated risk of developing acute thrombosis after LHP at RR of 5.50 (CI: 1.24–24.41, *p* = 0.02) through the pooled estimate of 4 studies (*n* = 279).

### Moderate-term outcome and complications

**Fig. 6:**
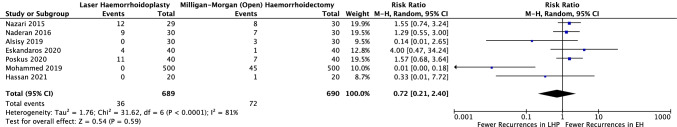
Forest plot of recurrence rate

Crucially, there was no significant difference between the rate of recurrence between the two groups from the pooled estimate of 7 studies (*n* = 1379), with a HR of 0.72 (CI: 0.21–2.40, *p* = 0.59), which tended to favour LHP (Fig. 6). Two studies utilised standardised patient questionnaires including the Wexner incontinence score, 36-item Short Form Health Survey (SF-36), and Faecal Incontinence Quality of Life (FIQOL). Poskus et al. reported no significant difference in the Wexner incontinence score and FIQOL between their LHP and CoH cohorts [45]. The SF-36, however, favoured CoH for the evaluation of General Health; while other components of the SF-36 were equivocal. However, LHP was evaluated as the best operation by patients. Shabahang et al. [46] result also favoured LHP at 6 months post-operation, where patients’ QoL was significantly better in their LHP cohort for all domains except for physical functioning.

Moderate-term complication rates were overall lower post-LHP, with a HR of 0.07 (CI: 0.02–0.31, *p* = 0.0004) for developing anal stenosis for patients undergoing LHP compared to CoH through the pooled estimate of 4 studies (*n* = 1240). Other moderate-term complications were not widely reported; one study described the incidence of anal stricture, with one patient from their CoH cohort and none after LHP. Two studies reported 8 total patients developing incontinence after CoH whilst all patients retained continence after LHP.

## Discussion

LHP is relatively novel and has previous evidence demonstrating good efficacy and tolerability. Consequently, a pair-wise meta-analysis comparing LHP against CoH was conducted as the authors deemed the direct comparison against the established gold standard was the most appropriate for the objective evaluation of LHP. Apart from pre-operative itching, the two groups had congruent baseline characteristics. All haemorrhoidal disease grades were included as only 2.4% of the cohort presented with grade IV. Therefore, whilst its more severe symptomology may interfere with outcomes such as pain, the small percentage bears minimal impact upon the meta-analysis. A deliberate choice to forgo meta-regression was made to reflect how both surgeries perform in real-world settings, where multiple confounders affect the patient’s recovery and postoperative pain, and interpretation of isolated post-regression data may not accurately reflect practical patient care. Given the paucity of literature comparing long-term (> 1 year) recurrence rates after LHP and CoH and clinical significance of postoperative pain, postoperative pain was chosen as the primary outcome.

### Pain and quality of life

In the current study, LHP was found to have resulted in significantly lower postoperative pain compared to CoH in the immediate period up to the first postoperative month, where the highest limitation in function and QoL occurs. Moreover, there was overall reduced analgesia use post-LHP compared to CoH, with reduced mean dosage and shorter duration of post-discharge oral analgesia use. Our findings are consistent with existing literature, where reduced pain is the greatest benefit of LHP [14, 32]. Importantly, pain is not limited to discomfort, but its effects expand into a myriad of sequelae associated with increased morbidity and mortality [47, 48].

Patients who underwent LHP experienced better short-term postoperative QoL, evidenced by quicker recovery and qualitative evaluation. However, it is challenging to ascertain the exact difference in recovery due to inter-study postoperative recovery time definition variation. Taken in context with decreased postoperative pain and risk of short-term complications, our findings suggest that LHP is superior in the short term.

### Efficacy and safety profile of LHP

A major determinant of CoH as the standard of care is its low postoperative recurrence. Crucially, there was no significant difference between recurrence of LHP and CoH in the moderate term. Alternative surgeries such as stapled haemorrhoidectomy and Doppler-guided transanal haemorrhoid artery ligation (HAL) had higher rates of recurrence than CoH [14, 49–51]. Therefore, whilst several of these options provided similar benefits to LHP over CoH, patients had to weigh these advantages against elevated recurrence risk. Unsurprisingly, our pooled mean follow-up duration was relatively short at 8.58 ± 9.55 months, as HD patients are not followed-up beyond a year due to the quick postoperative healing time [22]. While a sub-year follow-up length is not ideal for assessing long-term outcomes, the clinical benefit of a long-term review is outweighed by unnecessary consumption of healthcare resources. Therefore, our findings remain an adequate and realistic reflection of the postoperative recurrence seen in routine clinical practice.

For providers, LHP provided reduced operative duration and intraoperative blood loss with no significant difference in the hospitalisation duration. From the patients’ perspective, risks of developing short-term complications after LHP, except acute thrombosis, which is expected considering LHP induces thrombosis, were either equivalent to or lower than CoH. Whilst not significant (*p* = 0.07), CoH had higher rates of ARU, which may predispose to further complications, including delirium, which burdens the patient-caregiver complex [52]. The risks of developing moderate-term complications were similarly lower post-LHP, though the studies did not extensively describe these likely due to the low risk inherent in these minor procedures.

### Comparison of LHP against other modalities

Postoperative pain and postoperative complications are the most concerning disadvantages of CoH and closed (Ferguson) haemorrhoidectomy [50, 53, 54]. Akin to LHP, alternative modalities including LigaSure, harmonic scalpel haemorrhoidectomy, stapled haemorhoidopexy, and HAL have better intraoperative and postoperative profiles compared to CoH and closed haemorrhoidectomy. Most importantly, they are similarly less painful for the patients. Crucially, these advantages were accompanied by the major issue of higher recurrence rates [14, 22, 49–51]. Therefore, given equivalent recurrence rate between LHP and CoH, evidenced by consistent results from independent institutions across 9 countries, LHP may potentially be superior to CoH by providing greater tolerability whilst maintaining CoH’s standard-defining efficacy in the moderate term [44–46, 54–62].

A noteworthy disadvantage of LHP is cost efficacy. Due to the technology’s novelty, the cost of the laser diode and generator are greater than consumables used in simpler procedures such as CoH, closed haemorrhoidectomy or HAL, which has been touted as a quick and affordable alternative [63, 64]. Currently, there is limited literature analysing LHP’s cost-effectiveness to support its widespread use globally. Consequently, further cost-analyses need to be conducted considering the interest of different stakeholders, including the patient, institutions, and insurance companies. The effects of variations of healthcare payment structures and distinct economical capacity of different countries on the cost-analysis of LHP should be recognised. Therefore, LHP’s uptake should be considered within national contexts.

It should be emphasised that every individual modality in the modern surgical arsenal for haemorrhoid management has its own unique surgical profile accompanied by its respective benefits and risks, the details of which lie beyond the scope of this review. Ultimately, despite the presence of CoH as the standard of care, there exist a plethora of favourable techniques widely available across institutions. In the fraternity’s bid for personalised surgical practice, an open discussion between patients and physicians is imperative to guide individualised care, catering to each patient’s unique goals and lifestyle.

### Strengths and limitations

The strength of this study was its ability to overcome inter-study variation and mitigate the effect of practical confounding factors, given the general paucity of studies comparing LHP and CoH directly. Our study fills an essential gap in the literature, providing a high level of evidence for a quick, effective, and tolerable procedure to treat a remarkably prevalent condition.

However, our study has limitations which should be considered during interpretation. The principal limitation was the inclusion of nonrandomised comparative cohort studies. Whilst we acknowledged that observational cohort studies are subjected to selection and observational bias, ultimately affecting the study’s evidence level, their inclusion was maintained to improve external validity as the larger sample size would provide a more generalisable assessment of our findings, mitigating the compromise on the study’s validity had CCSs been excluded. It should also be noted that the exact laser probe used differed across studies, though the procedure is broadly similar. Biolitec^©^ provides the laser equipment for one RCT (Poskus et al.), though the company was not involved in the study design and data analysis [45]. We acknowledge that the higher cost of LHP’s equipment may be a bias in the included studies; however, as the methodological quality of the included studies was found to be adequate, its impact on the final pooled analysis may be minimal.

Most of our included studies hail from Eastern European and Middle Eastern nations, owing to the paucity of available studies from larger territories such as North America or East Asia. It should be noted that they had limited follow-up periods, where larger studies such as the eTHoS trial measure symptom recurrence up to 24 months post-intervention, therefore disallowing the evaluation of long-term post-procedure recurrence rate of LHP [65, 66]. Nevertheless, all RCTs and CCSs on the subject were included, with the evaluation of moderate-term efficacy. Our findings should encourage Western institutions to increase their attention to LHP. Additionally, the results were tabulated from a limited sample size of twelve studies. However, the funnel plot complemented by the results of Egger’s test pointed to low possibility of publication bias.

## Conclusion

This systematic review revealed a paucity of well-conducted, large RCTs comparing CoH and LHP that measure symptom recurrence as a primary outcome and a minimum follow-up period of 24 months. Our findings suggest that LHP provides benefits firstly to patients through reduced intraoperative blood loss, substantial reduction of postoperative pain up till the first postoperative month, reduced risk of most postoperative short- and moderate-term complications and improved postoperative QoL; and secondly to providers by reducing operative time. Crucially, the moderate-term recurrence rate is equivalent to CoH. Our findings contribute to the pool of evolving data regarding contemporary surgical treatments for HD. In addition, cost-utility data in a robust health economics study is needed.

## Supplementary Information

Below is the link to the electronic supplementary material.

Supplementary file1 (PDF 383 KB)

Supplementary file2 (PNG 646 KB)

Supplementary file3 (PDF 216 KB)

Supplementary file4 (PDF 220 KB)

Supplementary file5 (PDF 176 KB)

Supplementary file6 (PDF 256 KB)

Supplementary file7 (PNG 646 KB)

Supplementary file8 (PDF 183 KB)

Supplementary file9 (PDF 196 KB)

Supplementary file10 (PDF 249 KB)

Supplementary file11 (PDF 249 KB)

Supplementary file12 (PDF 261 KB)

Supplementary file13 (PDF 252 KB)

Supplementary file14 (PDF 267 KB)

Supplementary file15 (PNG 515 KB)

Supplementary file16 (PNG 133 KB)

Supplementary file17 (PNG 160 KB)

Supplementary file18 (PNG 42 KB)

Supplementary file19 (PDF 166 KB)
