# Supplementary figures and images for: Systematic review and meta-analysis of postoperative pain and symptoms control following laser haemorrhoidoplasty versus Milligan-Morgan haemorrhoidectomy for symptomatic haemorrhoids: a new standard
Source: Int J Colorectal Dis. 2022 Jul 29;37(8):1759–71. doi: 10.1007/s00384-022-04225-4 (PMC9388431; doi:10.1007/s00384-022-04225-4)

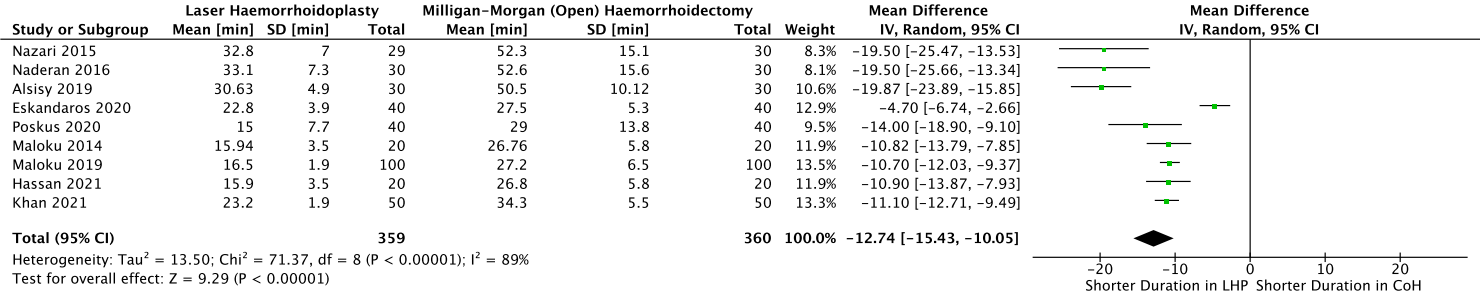

Supplement: Supplementary file 1 — Supplementary file1 (PDF 383 KB) [file 384_2022_4225_MOESM1_ESM.pdf]

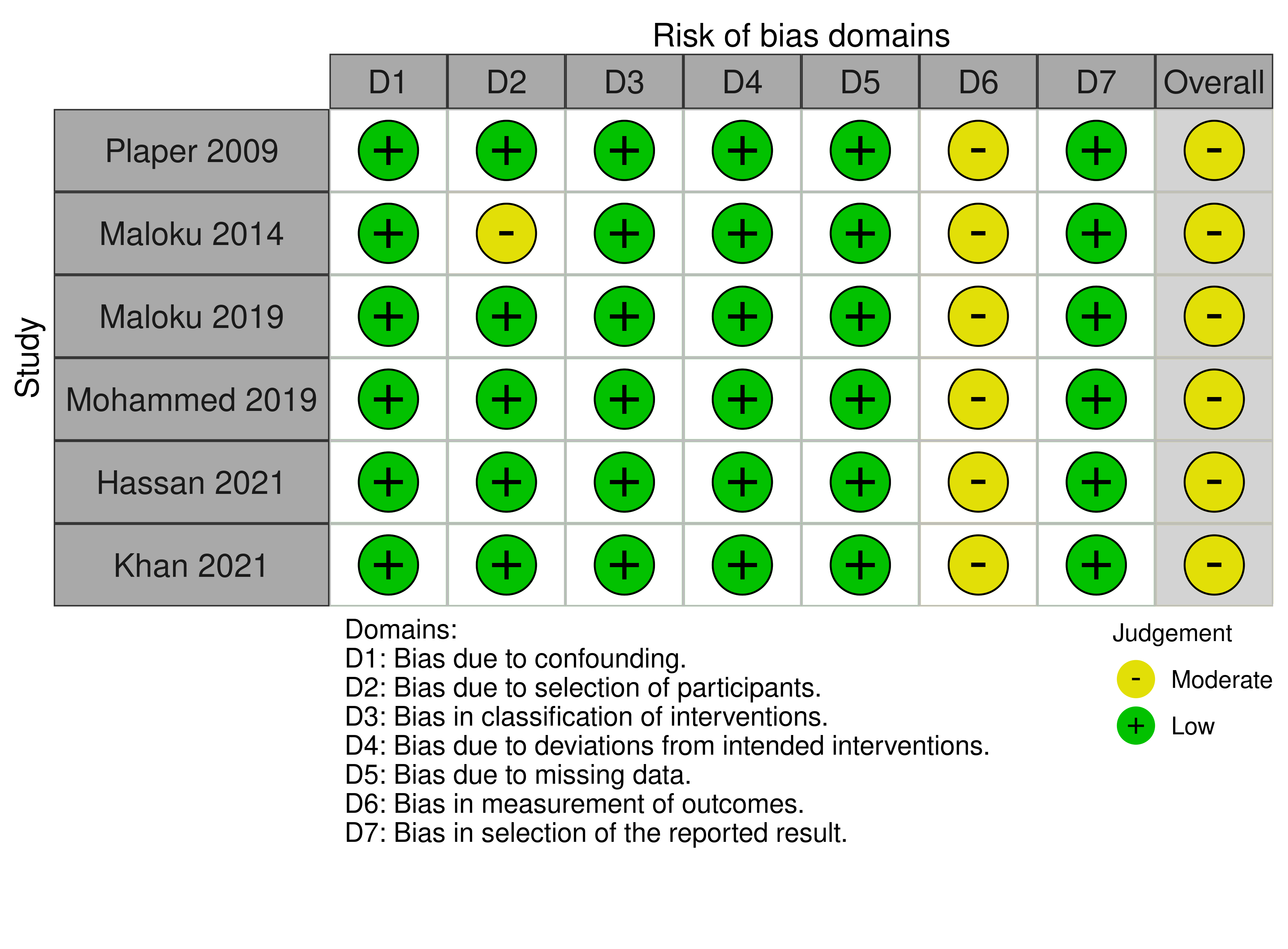

Supplement: Supplementary file 2 — Supplementary file2 (PNG 646 KB) [file 384_2022_4225_MOESM2_ESM.png]

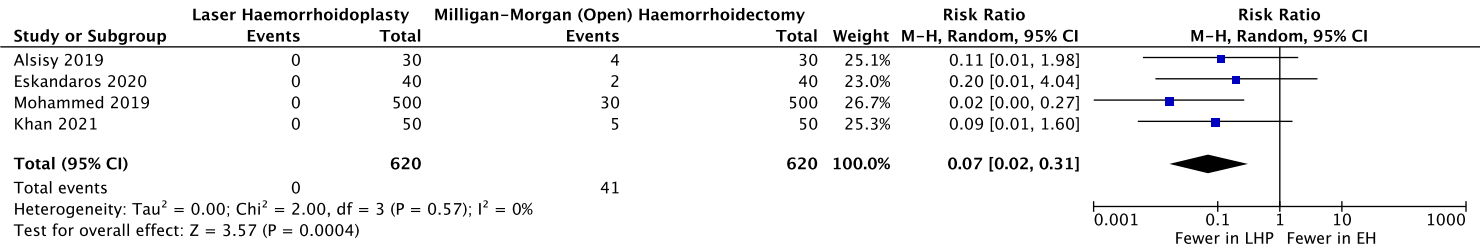

Supplement: Supplementary file 3 — Supplementary file3 (PDF 216 KB) [file 384_2022_4225_MOESM3_ESM.pdf]

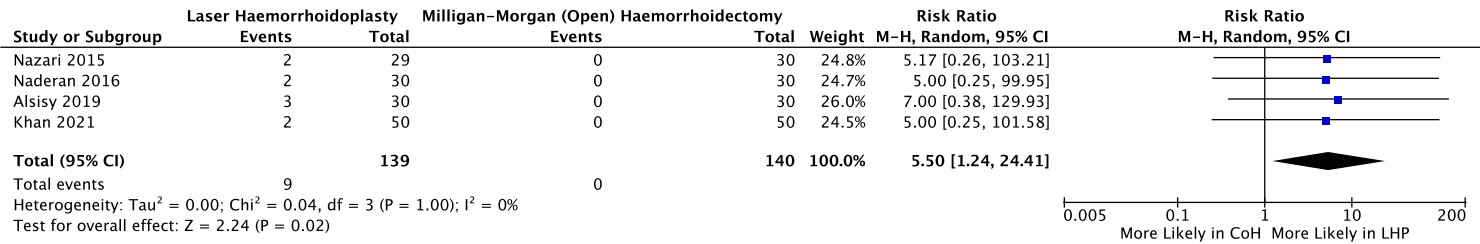

Supplement: Supplementary file 4 — Supplementary file4 (PDF 220 KB) [file 384_2022_4225_MOESM4_ESM.pdf]

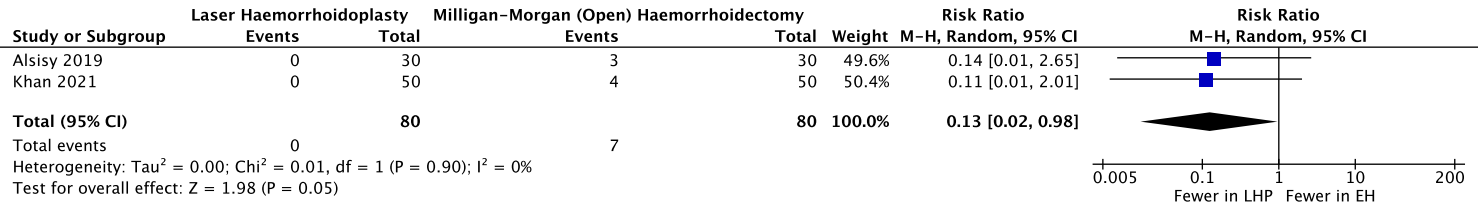

Supplement: Supplementary file 5 — Supplementary file5 (PDF 176 KB) [file 384_2022_4225_MOESM5_ESM.pdf]

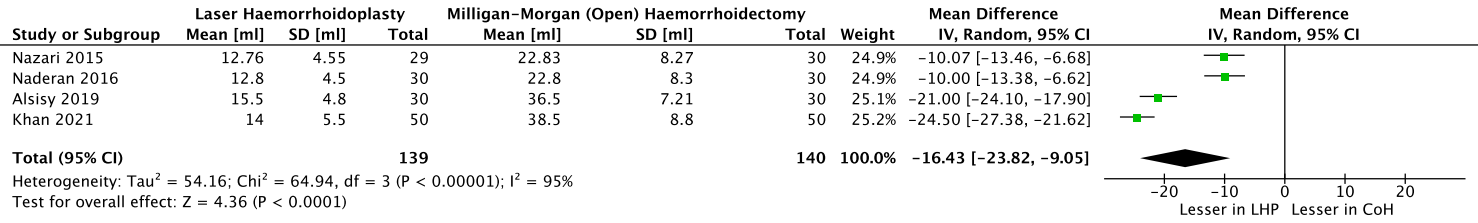

Supplement: Supplementary file 6 — Supplementary file6 (PDF 256 KB) [file 384_2022_4225_MOESM6_ESM.pdf]

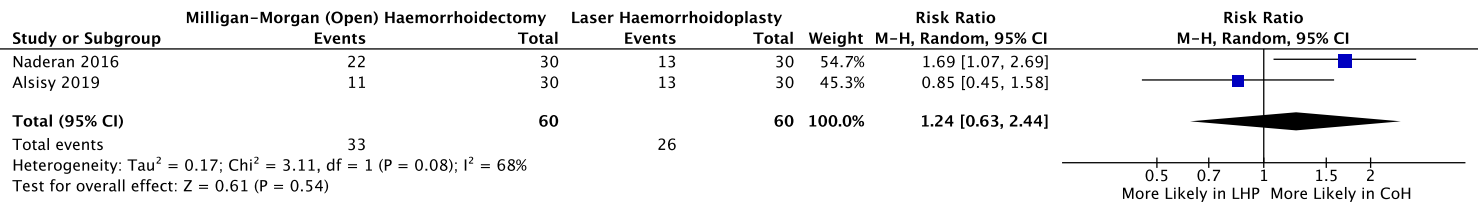

Supplement: Supplementary file 8 — Supplementary file8 (PDF 183 KB) [file 384_2022_4225_MOESM8_ESM.pdf]

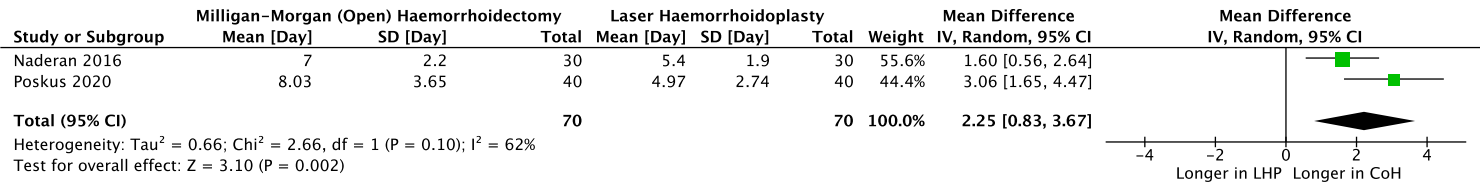

Supplement: Supplementary file 9 — Supplementary file9 (PDF 196 KB) [file 384_2022_4225_MOESM9_ESM.pdf]

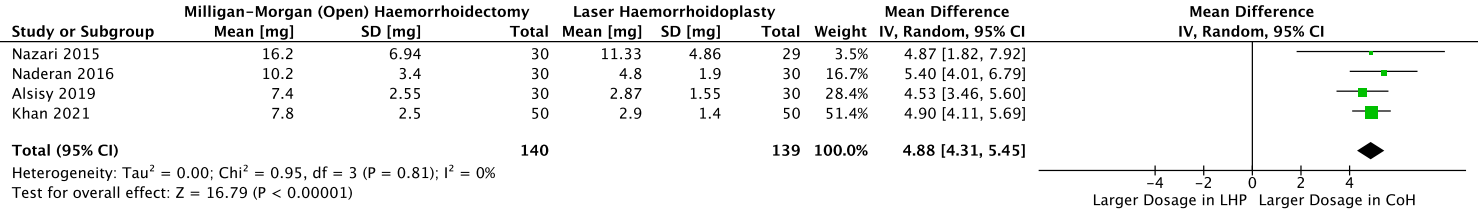

Supplement: Supplementary file 10 — Supplementary file10 (PDF 249 KB) [file 384_2022_4225_MOESM10_ESM.pdf]

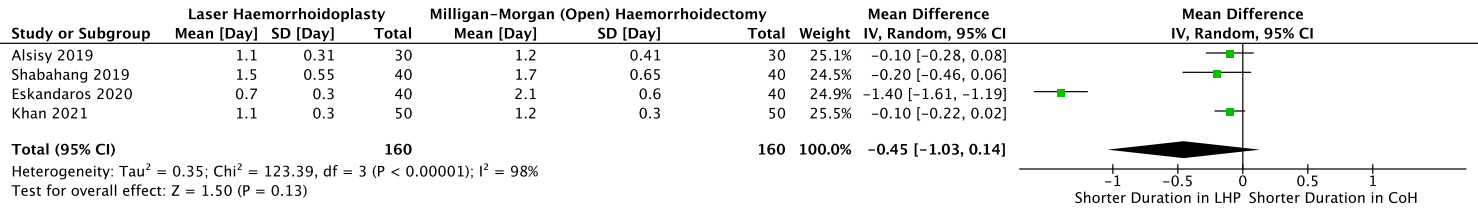

Supplement: Supplementary file 11 — Supplementary file11 (PDF 249 KB) [file 384_2022_4225_MOESM11_ESM.pdf]

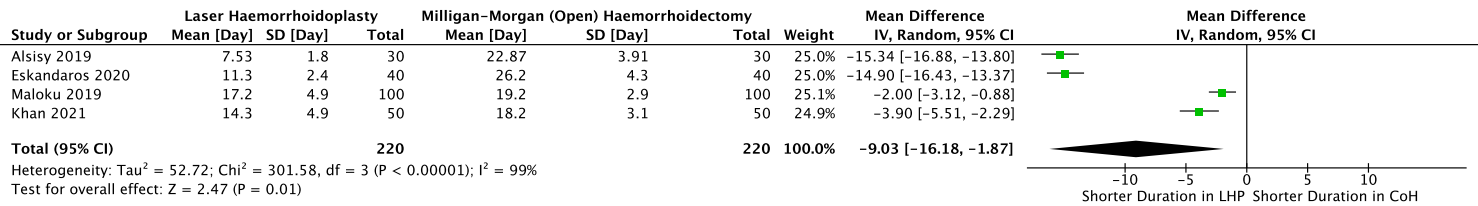

Supplement: Supplementary file 12 — Supplementary file12 (PDF 261 KB) [file 384_2022_4225_MOESM12_ESM.pdf]

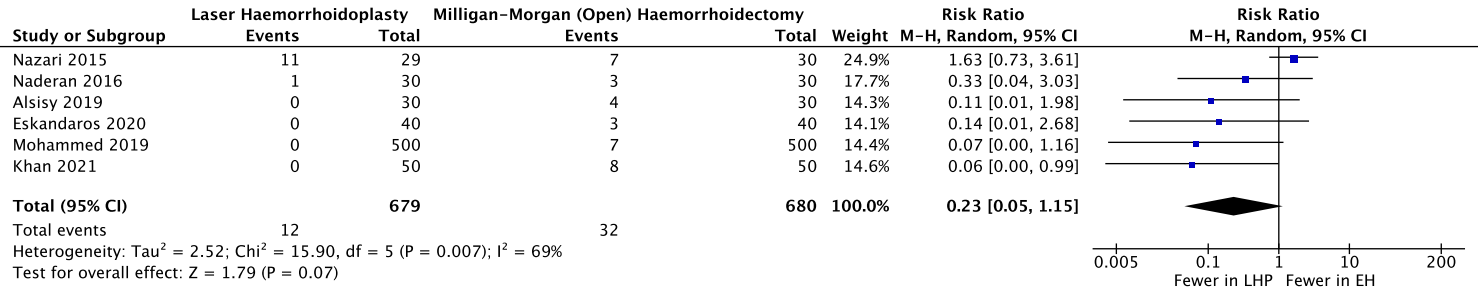

Supplement: Supplementary file 13 — Supplementary file13 (PDF 252 KB) [file 384_2022_4225_MOESM13_ESM.pdf]

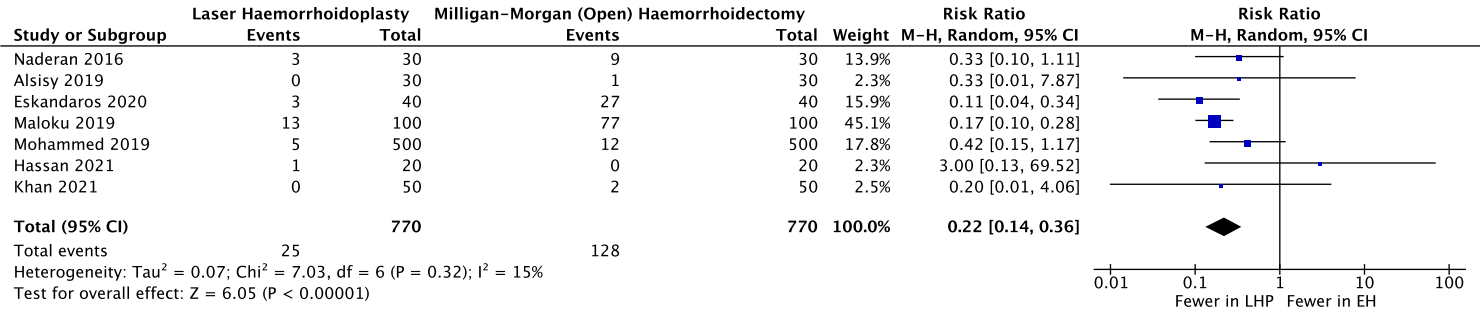

Supplement: Supplementary file 14 — Supplementary file14 (PDF 267 KB) [file 384_2022_4225_MOESM14_ESM.pdf]

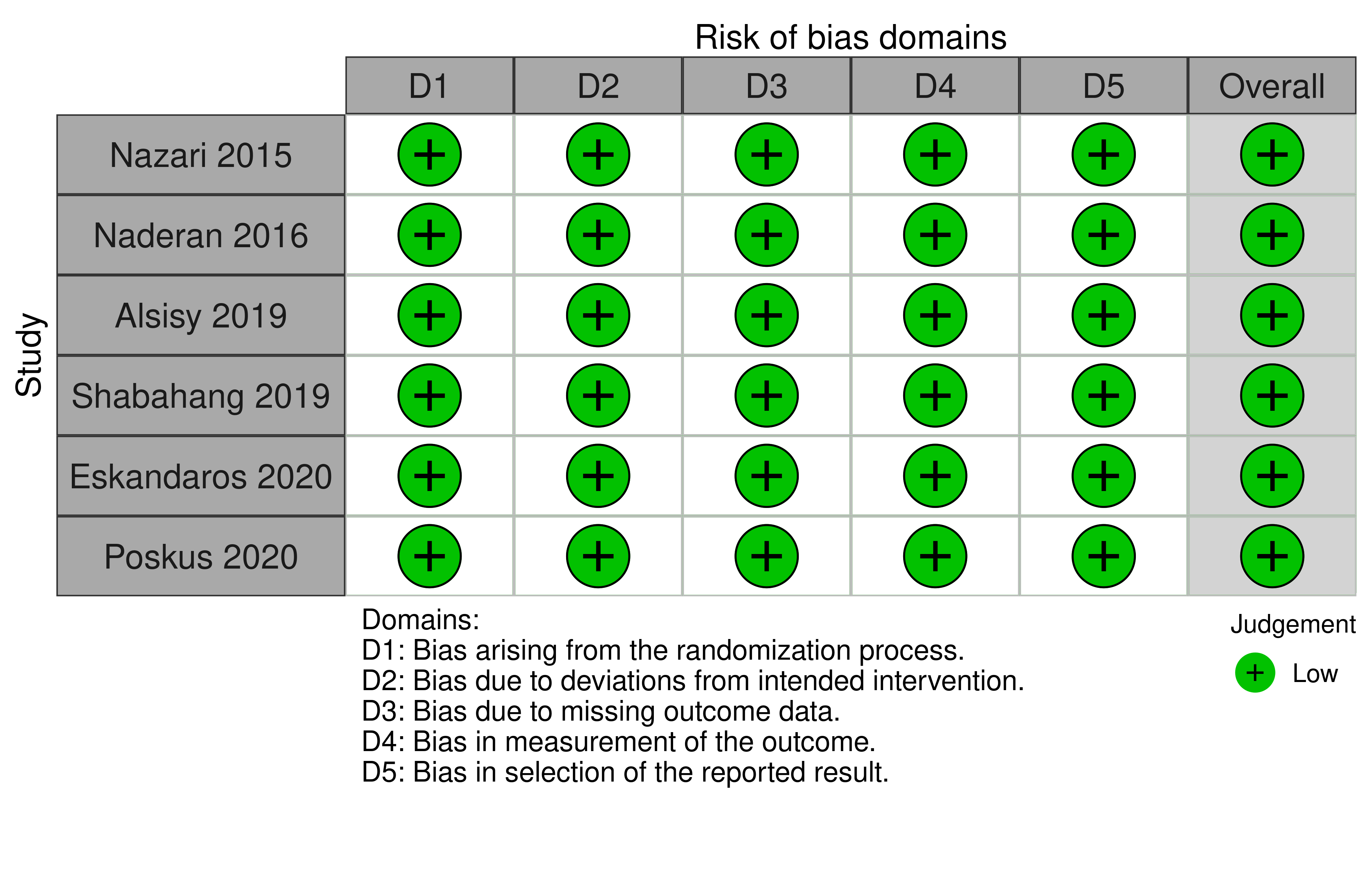

Supplement: Supplementary file 15 — Supplementary file15 (PNG 515 KB) [file 384_2022_4225_MOESM15_ESM.png]

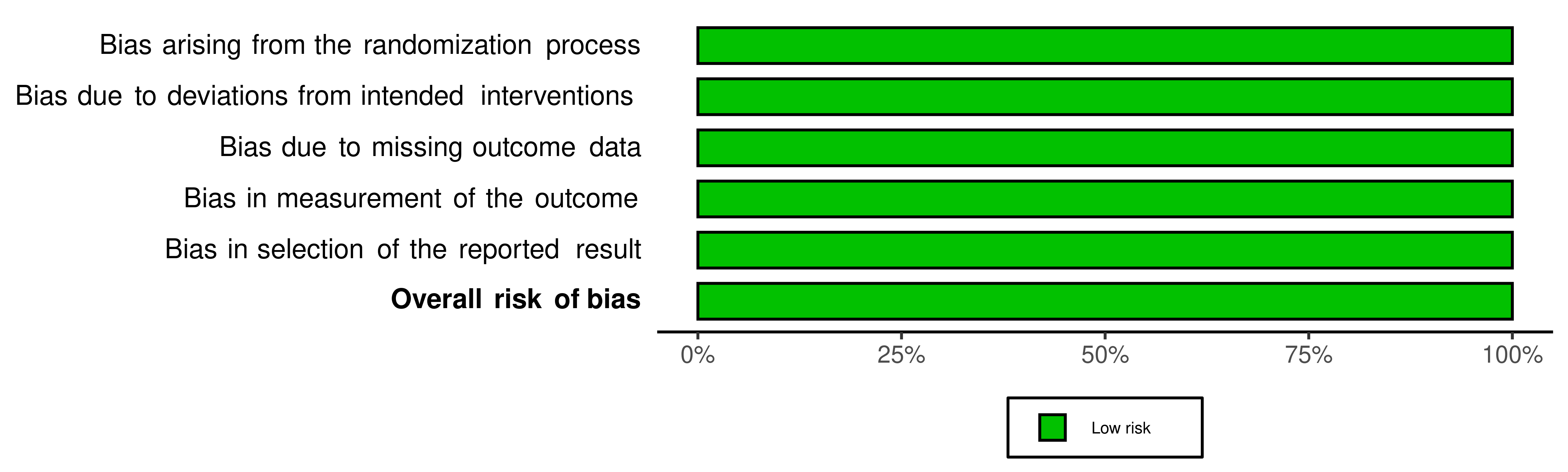

Supplement: Supplementary file 16 — Supplementary file16 (PNG 133 KB) [file 384_2022_4225_MOESM16_ESM.png]

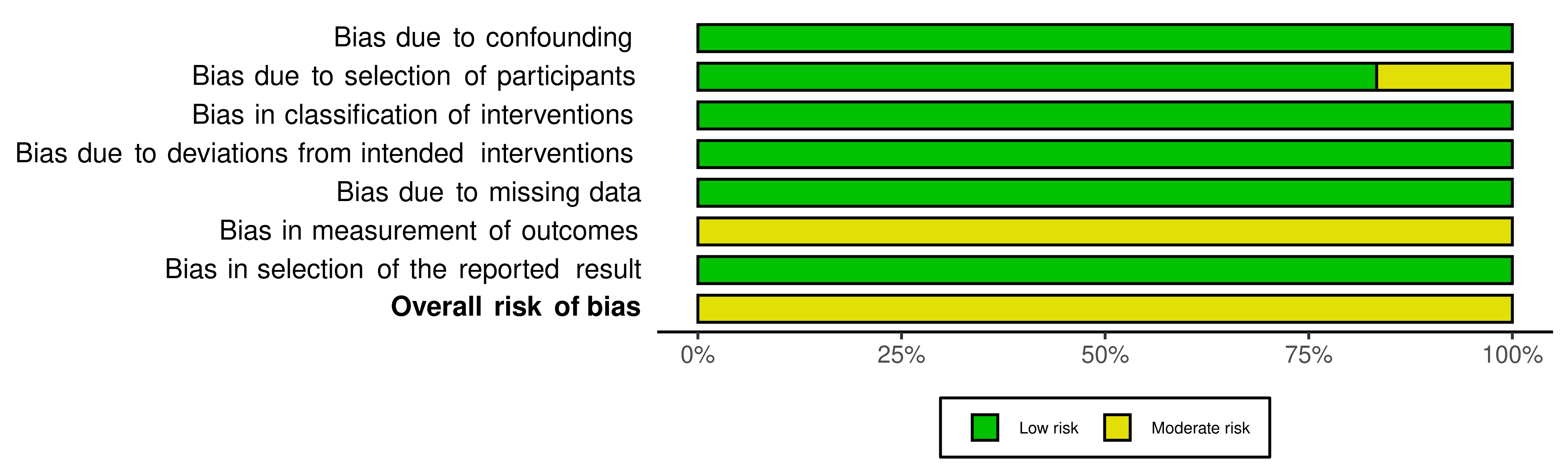

Supplement: Supplementary file 17 — Supplementary file17 (PNG 160 KB) [file 384_2022_4225_MOESM17_ESM.png]

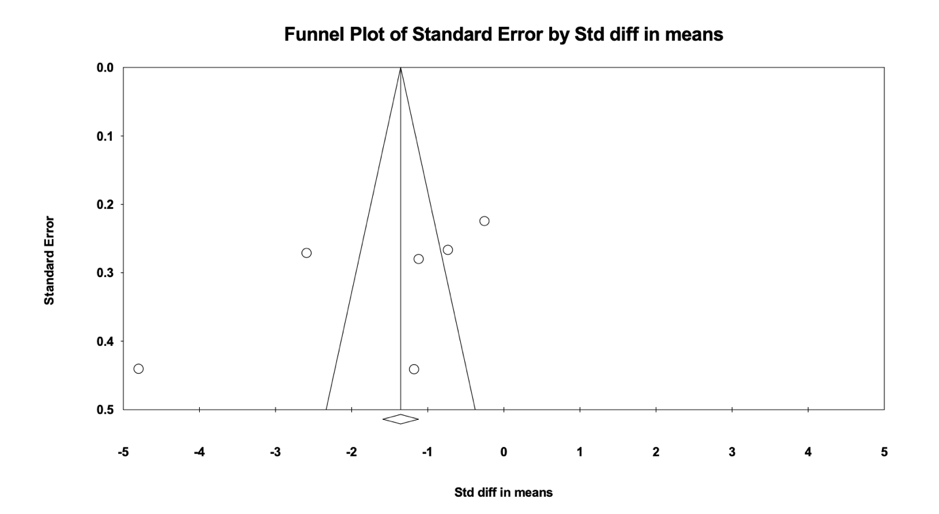

Supplement: Supplementary file 18 — Supplementary file18 (PNG 42 KB) [file 384_2022_4225_MOESM18_ESM.png]
