# Supplementary material for: Systematic review and meta-analysis of postoperative pain and symptoms control following laser haemorrhoidoplasty versus Milligan-Morgan haemorrhoidectomy for symptomatic haemorrhoids: a new standard
Source: Int J Colorectal Dis. 2022 Jul 29;37(8):1759–71. doi: 10.1007/s00384-022-04225-4 (PMC9388431; doi:10.1007/s00384-022-04225-4)
Supplement: Supplementary file 19 — Supplementary file19 (PDF 166 KB) [file 384_2022_4225_MOESM19_ESM.pdf]

Egger's regression intercept

Intercept	-11.78187
Standard error	7.10436
95% lower limit (2-tailed)	-31.50674
95% upper limit (2-tailed)	7.94300
t-value	1.65840
df	4.00000
P-value (1-tailed)	0.08629
P-value (2-tailed)	0.17258
